# Implementation of ubiquitous chromatin opening elements as artificial integration sites for CRISPR/Cas9‐mediated knock‐in in mammalian cells

**DOI:** 10.1002/elsc.202200047

**Published:** 2023-03-09

**Authors:** Seul Mi Kim, Jaejin Lee, Jae Seong Lee

**Affiliations:** ^1^ Department of Molecular Science and Technology Ajou University Suwon Republic of Korea

**Keywords:** Chinese hamster ovary (CHO), CRISPR/Cas9, hot spot, knock‐in, ubiquitous chromatin opening element

## Abstract

CRISPR/Cas9‐mediated targeted gene integration (TI) has been used to generate recombinant mammalian cell lines with predictable transgene expression. Identifying genomic hot spots that render high and stable transgene expression and knock‐in (KI) efficiency is critical for fully implementing TI‐mediated cell line development (CLD); however, such identification is cumbersome. In this study, we developed an artificial KI construct that can be used as a hot spot at different genomic loci. The ubiquitous chromatin opening element (UCOE) was employed because of its ability to open chromatin and enable stable and site‐independent transgene expression. UCOE KI cassettes were randomly integrated into CHO‐K1 and HEK293T cells, followed by TI of enhanced green fluorescent protein (EGFP) onto the artificial UCOE KI site. The CHO‐K1 random pool harboring 5′2.2A2UCOE‐CMV displayed a significant increase in EGFP expression level and KI efficiency compared with that of the control without UCOE. In addition, 5′2.2A2UCOE‐CMV showed improved Cas9 accessibility in the HEK293T genome, leading to an increase in indel frequency and homology‐independent KI. Overall, this assessment revealed the potential of UCOE KI constructs as artificial integration sites in streamlining the screening of high‐production targeted integrants by mitigating the selection of genomic hot spots.

**Abbreviations**
CHO: Chinese hamster ovary
CLD: cell line development
CMV: cytomegalovirus
DSB: double‐strand break
EGFP: enhanced green fluorescent protein
GOI: gene of interest
HA: homology arm
HDR: homology‐directed repair
HEK: human embryonic kidney
HITI: homology‐independent targeted insertion
KI: knock‐in
MFI: mean fluorescence intensity
NHEJ: non‐homologous end joining
PCR: polymerase chain reaction
PuroR: puromycin resistance gene
RI: random integration
RMCE: recombinase‐mediated cassette exchange
UCOE: ubiquitous chromatin opening element
USER: uracil‐specific excision reagent

## 1 INTRODUCTION

Mammalian cells are popular therapeutic production systems owing to their ability to produce complex glycosylated proteins. Most recombinant biotherapeutics are produced in Chinese hamster ovary (CHO) and human embryonic kidney (HEK) 293 cell lines [1, 2]. For large‐scale manufacturing of therapeutic proteins, the generation of stable cell lines with transgenes integrated into the genome is necessary. The traditional cell line development (CLD) method is based on random integration (RI) of expression cassettes containing the gene of interest (GOI) into the host genome. Transgenes are integrated into random loci with different chromatin structures, resulting in high levels of heterogeneous transgene expression and unexpected transgene silencing. Therefore, extensive effort and time are required to screen high and stable cell lines from RI pools. Targeted knock‐in (TI) has become feasible using genome editing tools, among which clustered regularly interspaced short palindromic repeats (CRISPR)/CRISPR‐associated protein 9 (Cas9) is the most popular method as it is easy to use through the simple design of a short single guide RNA (sgRNA). The TI approach using these tools can mitigate clonal variation and allow the prediction of transgene expression level, stability, and even knock‐in (KI) efficiency [3].

To fully implement TI‐mediated CLD, it is critical to identify genomic hot spots that are transcriptionally active loci and render high and stable transgene expression in mammalian cells. The underlying molecular and sequence‐specific mechanisms for hot spot activity are poorly understood; however, its expression properties clearly enhance transcriptional activity and stability. Most hot spots are located in gene‐rich chromosomal regions covered by euchromatin markers, whereas heterochromatin regions are highly correlated with expression silencing by promoter methylation, histone deacetylation, and histone methylation [4]. Moreover, the criteria for searching for novel hot spots are based on open chromatin markers [5]. Therefore, TI into the euchromatin locus is beneficial for the generation of high‐producing recombinant mammalian cell lines.

The open chromatin structure in the eukaryotic genome not only enhances gene expression but also increases the efficiency of CRISPR/Cas9 gene editing. As CRISPR/Cas9‐mediated KI suffers from low targeting efficiency, a high KI efficiency is important for facilitating TI‐mediated CLD. CRISPR/Cas9‐mediated KI methods rely on the DNA repair pathway, including the non‐homologous end joining (NHEJ) and homology‐directed repair (HDR) pathways when induced by double‐strand breaks (DSBs) at the target genomic sequence. Recent studies have shown that the euchromatin structure positively affects the binding and editing efficiency of Cas9 by increasing its chromatin accessibility [6], whereas Cas9 tends to inefficiently search target sites in heterochromatin regions [7]. Several studies aimed to enhance Cas9‐mediated DNA editing by opening heterochromatin structures with transcription activator‐Cas9 fusion or inhibition of heterochromatin‐associated proteins [8, 9].

Although hot spots are important for generating high‐producing recombinant cell lines, finding hot spots for each cell type is still challenging. Many researchers have applied several strategies to identify transcriptional hot spots, and some empirical studies have found new sites via lentiviral‐ and/or site‐specific recombinases‐ based integration mechanisms with or without next‐generation sequencing [10, 11, 12]. However, the rational identification of endogenous hot spots has limitations in the efficient prediction of hot spot activity, and empirical approaches to screen and identify chromosomal sites with desirable expression properties are time‐consuming.

**PRACTICAL APPLICATION**

In site‐specific integration‐based cell line development (CLD), the identification and validation of hot spots are needed for efficient and streamlined therapeutic protein production. We demonstrated that the introduction of chromatin opening elements, UCOE, with the cytomegalovirus (CMV) promoter into the host cell genome can be used as artificial integration sites. 5′2.2A2UCOE‐CMV randomly integrated sites improved CRISPR/Cas9‐mediated genome editing efficiency including knock‐in (KI) efficiency and indel frequency. In addition, the UCOE containing artificial constructs provide relatively high and stable transgene expression in Chinese hamster ovary (CHO) cells, demonstrating the potential of the UCOE KI constructs as a landing pad in streamlining the generation of high‐production targeted integrants.

To date, there have been no attempts to create hot spots. In this study, we aimed to generate ubiquitously active euchromatic regions in the genome for use as hot spots. To generate expression‐friendly, stable, and KI‐prone artificial integration sites, we utilized chromatin‐remodeling DNA elements, among which ubiquitous chromatin opening elements (UCOE) were adopted. UCOE is a chromatin remodeling DNA element derived from bidirected promoter sequences [13]. UCOE is characterized by methylation‐free CpG islands and euchromatic histone marks, causing the integration site to be resistant to the formation of a closed chromatin structure by DNA methylation and histone post‐translational modifications [14]. The most well‐known UCOE, A2UCOE, is derived from the human *HNRPA2B1‐CBX3* locus. A2UCOE makes juxtaposed promoters more active and stable for transgene expression at random integrated sites. Most importantly, A2UCOE‐linked transgenes integrated into the centromere or telomeres maintain expression for long culture [15].

We hypothesized that UCOE‐containing insertion sites might be highly active and editing‐prone, generally shown in hot spots in mammalian cell lines. To confirm this hypothesis, we generated artificial KI constructs harboring cytomegalovirus (CMV) promoter, A2UCOE, or A2UCOE‐CMV and applied a split GFP system to measure HDR‐mediated KI. To compare the potential of each construct at various sites, we measured genome editing efficiency, transgene expression levels, and stability at RI sites in CHO‐K1 and HEK293T cells.

## 2 MATERIALS AND METHODS

### 2.1 Plasmid construction

All donor plasmids and sgRNA/Cas9 expression plasmid vectors used in this study are listed in Supplementary Table S1. All donor plasmids, except homology‐independent targeted insertion (HITI) donors, were cloned via the uracil‐specific excision reagent (USER) cloning method.

Different ranges of A2UCOE fragments were obtained from HEK293T genomic DNA with different primer pairs using Phusion Hot Stat II DNA polymerase (F549L, Thermo Fisher Scientific, Waltham, MA). For RI of artificial KI constructs or EGFP RI donors, CMV, CMV‐EGFP_1‐10_, CMV‐EGFP, EGFP_1‐10_, and T2A‐PuroR‐BGHpA fragments were amplified from pEGFP‐C1 (Clontech, Palo Alto, CA) or AAVS1‐eGFP donors [16]. All USER bricks were generated by USER polymerase chain reaction (PCR) using internal‐deoxyuridine containing primers and Phusion U Hot Start DNA Polymerase (F555L, Thermo Fisher Scientific).

HITI donors were constructed using the Golden Gate Cloning method. For T2A‐TagRFP657‐BGHpA HITI donor, sgEGFP_1‐10_‐T2A, BGHpA‐sgEGFP_1‐10_ fragments were amplified using primers, including the sgEGFP_1‐10_ sequence from AAVS1‐eGFP donor and TagRFP657 amplicons obtained from pTagRFP657‐N1 (#31959, Addgene). Plasmid fragments were generated using the Phusion High‐Fidelity PCR Master Mix with HF Buffer (F531L, Thermo Fisher Scientific) and ligated with Bbs1 restriction sites on both sides of each fragment.

The plasmid backbone for all donor plasmids were amplified from pcDNA3.1 (Thermo Fisher Scientific). Gel electrophoresis was then performed on 1% or 2% agarose Tris‐Acetate‐EDTA (TAE) gels, and PCR amplicons were purified using the NucleoSpin Gel and PCR Cleanup Kit (Macherey‐Nagel, Duren, Germany). Appropriate fragments and backbones were assembled into each plasmid vector using the USER enzyme (M5505L, New England Biolabs, Ipswich, MA) for USER cloning or Bbs1 (R0539L, NEB) and T4 DNA ligase (M0202L, NEB) for Golden Gate cloning. Two complementary oligonucleotides, including sgRNAs and overhang (Forward: CACCG‐N_20_, Reverse: AAAC‐N_20_‐C), were annealed and assembled into digested pU6‐(BbsI) CBh‐Cas9‐T2A‐mCherry (Addgene plasmid #64324) using T4 ligase (Thermo Fisher Scientific), resulting in the sgRNA‐Cas9 plasmid. The plasmid vectors were transformed into *E.coli* One Shot Mach1 competent cells (Thermo Fisher Scientific) and verified using Sanger sequencing. Purified plasmid vectors for transfection in HEK293T and CHO‐K1 cells were obtained using NucleoBond Xtra Midi EF (Macherey‐Nagel), according to the manufacturer's instructions.

### 2.2 Cell lines and culture maintenance

The CHO‐K1 cell line was maintained in Dulbecco's modified Eagle's medium (DMEM; Gibco, Gaithersburg, MD) supplemented with 10% (v/v) fetal bovine serum (FBS; Hyclone, Logan, UT). HEK293T cells were grown in DMEM supplemented with 10% (v/v) FBS and 2 mM L‐glutamine (Hyclone). These cell lines were incubated at 37°C in a humidified 5% CO_2_ atmosphere and passaged every 3–4 days. Viable cell density and viability were measured using the trypan blue dye exclusion method and an automated cell counter (Countess II FL; Invitrogen, Carlsbad, CA).

### 2.3 Generation of random pools

A total of 1.0 × 10^6^ cells of CHO‐K1 were transfected with 10 µg of each plasmid donor for RI using a NEPA21 electroporator (Nepagene, Chiba, Japan). HEK293T cells were seeded at 0.2 × 10^6^ cells/mL in 6‐well cell culture plates with 3 mL culture medium. After approximately 24 h, the cells were transfected with 2.5 µg of each RI donor using Lipofectamine 3000 (L3000075, Invitrogen). After 2–3 days of transfection, transfected cells were seeded at 1.0 × 10^5^ cells/mL in T25 flasks (NUN‐156367, Thermo Fisher Scientific) containing 5 mL culture medium with 10 µg/mL for CMV‐ and A2UCOE‐CMV CHO‐K1, 6 µg/mL for A2UCOE CHO‐K1, 3 µg/mL for CMV‐ and A2UCOE‐CMV HEK293T, and 2 µg/mL puromycin (Sigma Aldrich, St. Louis, MO) for A2UCOE HEK293T cells. Cell pools were generated by puromycin selection for 2–3 weeks.

### 2.4 Tracking of Indels by DEcomposition (TIDE) analysis

Two days after transfection with sgEGFP_1‐10_/Cas9 vector or #64324 as a control, genomic DNA from cell pellets was purified using the GeneJET Genomic DNA Purification Kit (K0721, Thermo Fisher Scientific). PCR amplification was conducted using Phusion High‐Fidelity PCR Master Mix (F‐531L, Thermo Fisher Scientific) and primers targeting EGFP_1‐10_ and PuroR (Table S2). The PCR products were purified using the NucleoSpin Gel and PCR Cleanup Kit (Macherey‐Nagel) and sequenced using Sanger DNA sequencing. An online software [17] was used to calculate the indel frequency of CRISPR/Cas9, and the parameters were set to default values.

### 2.5 CRISPR/Cas9‐mediated KI

For HDR‐mediated KI, CHO‐K1 and HEK293T random pools harboring artificial KI constructs at random loci were seeded at 0.2 × 10^6^ cells/mL in 6‐well cell culture plates containing 3 mL cell culture media with appropriate puromycin concentration. After 24 h, the sgEGFP_1‐10_/Cas9 vector was co‐transfected with the EGFP_11_ HDR donor in a 1:1 ratio (w/w) using Lipofectamine 3000. Flow cytometry analysis was conducted on day 6 for CHO‐K1 cells and day 5 for HEK293T cells. For NHEJ‐mediated KI, sgEGFP_1‐10_/Cas9 vector and T2A‐TagRFP657‐BGHpA HITI donor were transfected at a 1:1 ratio (w/w) into CHO‐K1 random pools using a NEPA21 electroporator and HEK293T random pools using Lipofectamine 3000, as described in Section 2.3. Flow cytometry analysis was performed on day 6 for CHO‐K1 cells and day 8 for HEK293T cells.

### 2.6 Flow cytometry

To measure KI efficiency and enhanced green fluorescent protein (EGFP) or TagRFP657 expression levels, transfected cells (>0.5 × 10^6^) were harvested and resuspended in phosphate‐buffered saline supplemented with 10% FBS. Flow cytometry analysis was performed using FACSCalibur (Becton Dickinson, Franklin Lakes, NJ) or NovoCyte Flow Cytometer (Agilent Technologies, Santa Clara, CA). Live single cells were gated by forward scatter versus side scatter plots, and the fluorescence threshold was set to ∼0.1% based on non‐fluorescent cells (negative control). Flow cytometry data were analyzed using FlowJo (Tree Star, OR) and NovoExpress software (Agilent Technologies).

### 2.7 Statistical analysis

Statistical analysis was performed using a one‐way analysis of variance followed by Dunnett's multiple comparison test or unpaired *t*‐test. GraphPad Prism software (GraphPad Software, San Diego, CA) was used to calculate *p* values, and *p* < 0.05 was considered significant (*p < 0.05, **p < 0.01, ***p < 0.001, ****p < 0.0001).

## 3 RESULTS AND DISCUSSION

### 3.1 Construction of the artificial KI constructs harboring A2UCOE

**FIGURE 1:**
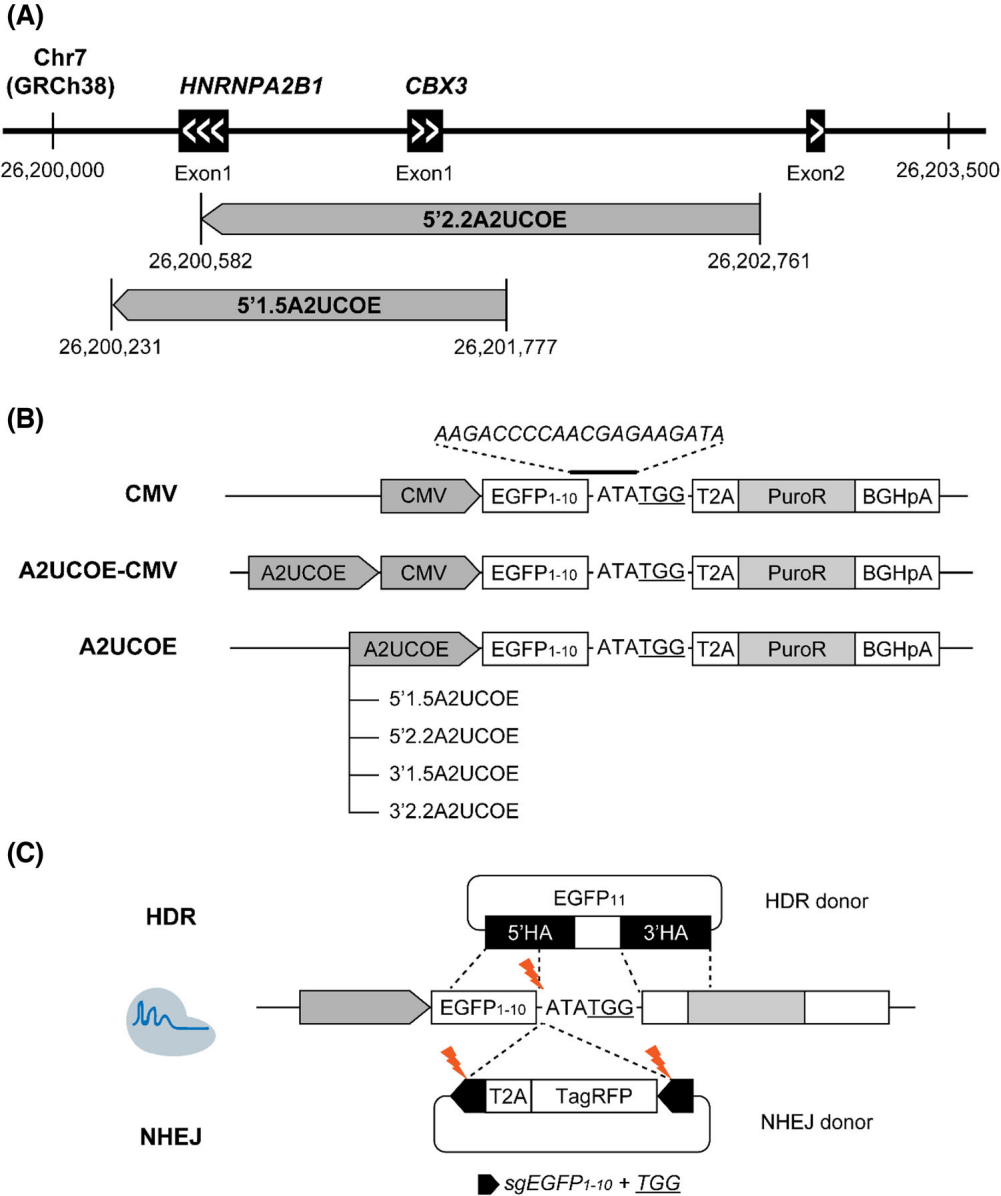
Schematic representation of HDR‐ and NHEJ‐mediated knock‐in (KI) in the UCOE random pools. (A) Illustration of the different A2UCOE fragments used in this study. 2.2 kb A2UCOE and 1.5 kb A2UCOE contain *CBX3* exon 1 and part or all sequence of *HNRNPA2B1* exon 1. Location indication is based on the human genome reference, GRCh38. (B) The construct for generating the CHO‐K1 and HEK293T random pools. The CMV promoter was used as a control element. Cas9 induces double‐strand break (DSB) directly after the EGFP_1‐10_ sequence. (C) Experimental design to evaluate HDR and NHEJ KI efficiency. HDR efficiency was measured by knocking‐in EGFP_11_ and completing the full EGFP sequence. NHEJ efficiency was evaluated with the HITI donor, including T2A‐TagRFP657. The underlined characters are PAM sequence and italic characters represent sgRNA target sequence. CMV, cytomegalovirus; EGFP, enhanced green fluorescent protein; HA, homology arm; HDR, homology‐directed repair; HITI, homology‐independent targeted insertion; NHEJ, non‐homologous end joining; PuroR, puromycin resistance gene (puromycin N‐acetyl‐transferase, pac); UCOE, ubiquitous chromatin opening element.

UCOE was hypothesized to improve KI efficiency and transgene expression level and stability when integrated into the host genome. To test this hypothesis, we designed an artificial KI construct containing A2UCOE (Figure 1A) and an exogenous sgRNA‐Cas9 target site. The KI construct vectors had a CMV, A2UCOE, or A2UCOE‐CMV promoter, followed by EGFP_1‐10_ sequence and puromycin resistance gene (PuroR) expression cassettes consisting of T2A‐PuroR‐BGHpA (Figure 1B). The CMV promoter was used as a control element as it is widely used in both CHO‐K1 and HEK293T for production owing to its high transcriptional activity; however, improvements are needed owing to its susceptibility to gene silencing by DNA methylation and histone deacetylation. The activity of A2UCOE is known to be orient‐dependent [18]. Therefore, we applied different ranges (1.5 or 2.2 kb) and 5′ or 3′ directions of A2UCOE with or without CMV to identify optimal fragments.

A split EGFP system was used to evaluate HDR‐mediated KI efficiency. The EGFP_1‐10_ sequence in artificial KI constructs encodes for 1–10 beta‐sheets of EGFP. Upon integration of another part of the split EGFP, EGFP_11_, via HDR‐mediated KI, the complete EGFP sequence was restored (Figure 1C). To implement the system with CRISPR/Cas9, we added an additional 3 bp and PAM (TGG) sequences after EGFP_1‐10_. As EGFP_1‐10_ and EGFP_11_ are non‐fluorescent on their own [19], they enable the detection of EGFP signals from KI cells only. In addition, this system can be utilized to measure the efficiency of the NHEJ‐based HITI [20]. The HITI donor includes sgRNA target sequences, same as the genomic target site, on both sides of the GOI. After Cas9 cleavage, some sgRNA sequences remained in the linearized donor and were integrated into the genomic target locus. To detect NHEJ‐mediated accurate KI efficiency, we designed the GOI in HITI donors as T2A‐TagRFP657‐BGHpA because T2A peptides are co‐translated with TagRFP657 when they keep in‐frame without indels in front of the GOI.

### 3.2 5′2.2A2UCOE‐CMV randomly integrated sites are the most active and genome editing‐prone in CHO‐K1 and HEK293T cells

To determine whether A2UCOE could increase transgene expression and CRISPR/Cas9‐mediated KI efficiency, we randomly integrated artificial KI constructs into CHO‐K1 and HEK293T cells. Cells were transfected with artificial KI constructs and then subjected to puromycin selection for approximately 2 weeks to generate random pool cells. Compared to strong CMV promoter‐based constructs (CMV and A2UCOE‐CMV), the 5′1.5A2UCOE HEK293T random pool and all 3′2.2A2UCOE random pools could not survive in the presence of puromycin, which might be due to the low inherent promoter activity of A2UCOE (data not shown).

**FIGURE 2:**
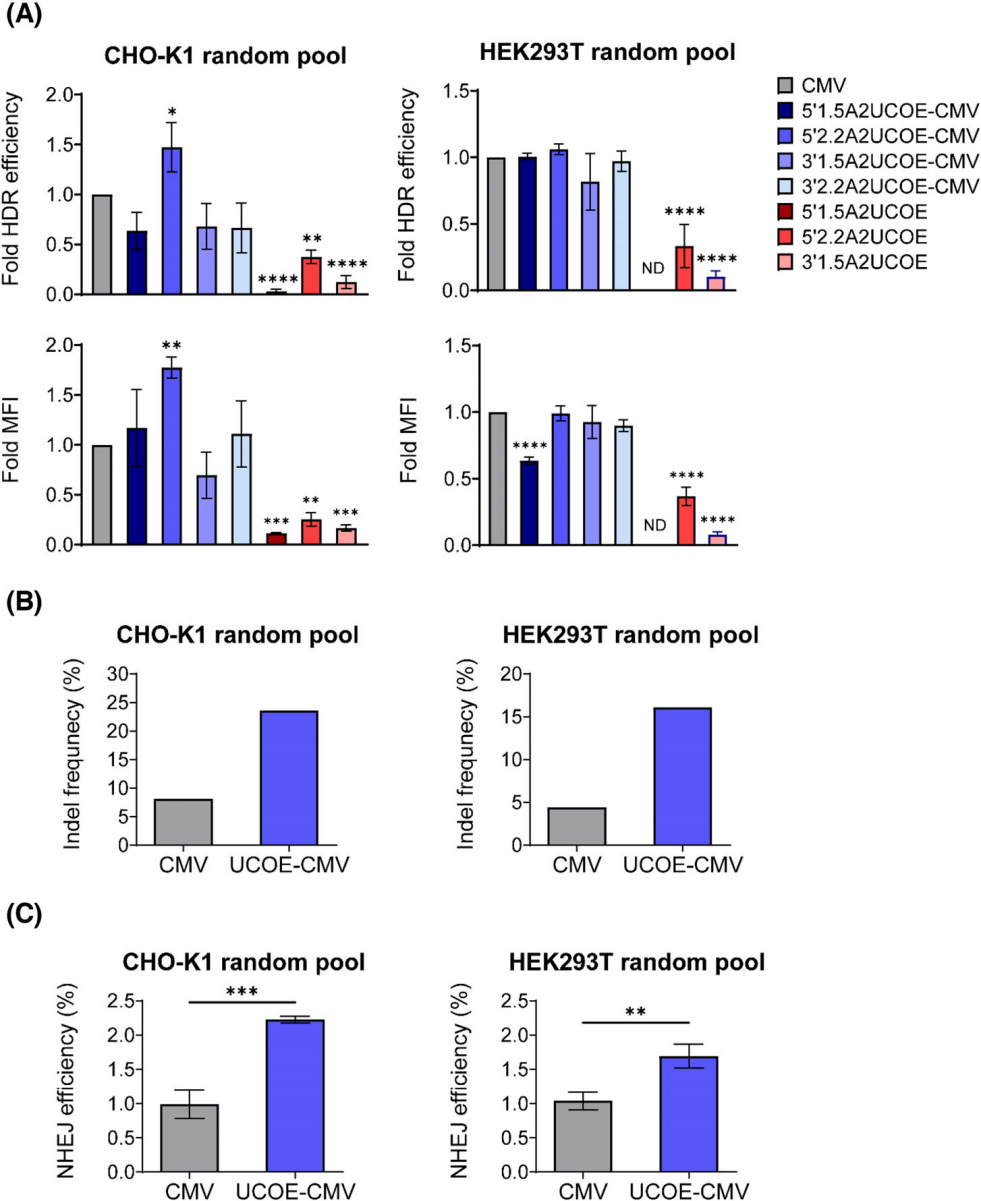
Targeted knock‐in (KI) efficiency and EGFP expression levels in random pools containing artificial KI constructs. (A) Fold HDR‐mediated KI efficiency and mean fluorescence intensity (MFI) of each construct compared to CMV in the CHO‐K1 and HEK293T random pools. Flow cytometry analysis was performed five days post‐transfection, and the relative MFI and percentage of EGFP^+^ populations are shown. (B) TIDE analysis of the UCOE insertion site at 2 days after transfection. (C) The NHEJ‐mediated KI efficiency at 6 days and 8 days after transfection in CHO‐K1 and HEK293T random pool. The error bars represent mean ± standard deviation of three independent experiments. *p ⩽ 0.05, **p ⩽ 0.01, ***p ⩽ 0.001, ****p ⩽ 0.0001 as determined by (A) ordinary one‐way or (C) unpaired *t*‐test. CMV, cytomegalovirus; EGFP, enhanced green fluorescent protein; ND, not detected; NHEJ, non‐homologous end joining; TIDE, Tracking of Indels by DEcomposition; UCOE, ubiquitous chromatin opening element.

To assess the transgene expression level and homology‐directed KI efficiency in all random pools in which artificial integration sites were integrated, the EGFP_11_ HDR donor plasmid was co‐transfected with the sgEGFP_1‐10_/Cas9 expression vector. EGFP signals were detected by flow cytometry on day 6, and the mean fluorescence intensity (MFI) and percentage of EGFP^+^ populations were determined (Figures 2A and S1). All CHO‐K1‐ and HEK239T‐derived A2UCOE random pools displayed significantly lower EGFP expression levels and HDR KI efficiencies than the CMV and A2UCOE‐CMV random pools. Among the A2UCOE random pools, CHO‐K1 and HEK293T random pools introduced with 5′2.2A2UCOE landing pads displayed the highest expression level and HDR KI efficiency (Figure 2A). Notably, the A2UCOE‐CMV random pools showed different results between CHO‐K1 and HEK293T cells. In CHO‐K1, only the 5′2.2A2UCOE‐CMV CHO‐K1 random pool showed a significant increase in both MFI and HDR KI efficiency, approximately 1.9‐ and 1.6‐fold, respectively, relative to the CMV CHO‐K1 random pool. On the other hand, A2UCOE‐CMV HEK293T random pools showed similar MFI and HDR KI efficiencies compared to the CMV HEK293T random pool, except 5′1.5A2UCOE‐CMV. These data suggest that 5′2.2A2UCOE is the most active A2UCOE fragment, and 5′2.2A2UCOE‐CMV randomly integrated sites could be used as effective landing pads, enabling improved HDR‐mediated TI and higher transgene expression than CMV in CHO‐K1 cells, but not in HEK293T cells.

To clearly verify the activity of 5′2.2A2UCOE, we transfected CHO‐K1 and HEK293T cells with EGFP RI donors harboring CMV or 5′2.2A2UCOE‐CMV and the full‐length EGFP sequence, followed by puromycin selection for random pool generation (Figure S2). Similar to the EGFP expression profiles shown in targeted integrants based on CHO‐K1 cells (Figure 2A), the EGFP population and MFI in 5′2.2A2UCOE‐CMV‐EGFP random pools was markedly higher than that in CMV‐EGFP random pools at the starting point, and this upward tendency was maintained for up to 10 passages. Consistent with previous studies demonstrating that stable cell lines made by RI of UCOE containing constructs did not show higher copy number than those made by UCOE‐free constructs [21, 22], the relative copy number of transgene was comparable between 5′2.2A2UCOE‐CMV‐EGFP and CMV‐EGFP random pools (Figure S3). This supports that the positive effects of 5′2.2A2UCOE are not derived from copy number variation. In HEK293T cells, the 5′2.2A2UCOE‐CMV‐EGFP random pools maintained EGFP expression during the 25 passages. However, the EGFP population and initial level of EGFP expression of 5′2.2A2UCOE‐CMV‐EGFP did not differ from that of CMV‐EGFP (Figure S2B and C). This result indicates that the 5′2.2A2UCOE fragment derived from HEK293T genome can successfully apply to CHO‐K1, but have a limited activity when combining with CMV promoter in HEK293T. In addition, 5′2.2A2UCOE‐CMV randomly integrated sites can serve as artificial integration sites, leading to high transgene expression levels similar to the expression levels observed in randomly generated stable cell lines.

Previous studies demonstrated that artificial open chromatin might induce the NHEJ pathway rather than the HDR pathway [23]. As the CMV promoter is strongly active in HEK293T cells, we assumed that there is a limit to the relative increase in HDR KI efficiency in the 5′2.2A2UCOE‐CMV HEK293T random pool. To evaluate whether the NHEJ repair pathway is more active in the 5′2.2A2UCOE‐CMV random pool than the CMV random pools, we compared NHEJ‐mediated knockout and KI efficiencies, as measured by indel frequency using TIDE analysis and the percentage of TagRFP657^+^ populations shown in Figure 1C, between CMV and 5′2.2A2UCOE‐CMV random pools. Interestingly, 5′2.2A2UCOE‐CMV showed 2.9‐ and 3.7‐fold increases in indel frequencies compared to CMV in the CHO‐K1 and HEK293T random pools, respectively (Figure 2B). This finding indicates that random 5′ 2.2A2UCOE‐CMV integration sites are more prone to DSBs in CHO‐K1 and HEK293T cells. Thereafter, we performed NHEJ‐mediated KI of artificial KI constructs with HITI donors in CMV and 5′2.2A2UCOE‐CMV random pools. HITI donors were co‐transfected with sgEGFP_1‐10_/Cas9 expression vectors, and TagRFP657 signals were detected by flow cytometry on days 6 and 8 in CHO‐K1 and HEK293T cells, respectively. Consistent with the knockout efficiencies, NHEJ KI efficiency was considerably higher in 5′2.2A2UCOE‐CMV in both the CHO‐K1 and HEK293T random pools (Figure 2C). Taken together, we demonstrated that 5′2.2A2UCOE‐CMV randomly integrated sites improved overall Cas9‐mediated genome editing efficiencies and transgene expression levels of targeted integrants.

## 4 CONCLUDING REMARKS

In site‐specific integration‐based CLD, the identification and validation of hot spots are needed for efficient and streamlined therapeutic protein production. However, the development of genomic hot spots requires excessive time and resources owing to limited information on hot spot hallmarks and difficulties in predicting the capability of identified sites. In this study, the introduction of chromatin opening elements, UCOE, with the CMV promoter sequences into the host cell genome was found to serve as artificial integration sites.

5′2.2A2UCOE‐CMV randomly integrated sites showed improved HDR‐mediated TI and higher transgene expression than CMV in a cell type‐dependent manner. Higher indel frequency and NHEJ‐mediated TI efficiency in 5′2.2A2UCOE‐CMV indicate that 5′2.2A2UCOE‐CMV improved Cas9 accessibility, ultimately leading to higher CRISPR/Cas9‐mediated genome editing efficiency, despite a limit to the increase in expression level in HEK293T, which might be due to the very high activity of the CMV promoter [24]. To expand the application of artificial KI constructs harboring A2UCOE at specific loci, further investigation in other cell types and various loci with diverse genomic contexts is needed.

In conclusion, we demonstrated that artificial KI constructs harboring A2UCOE improve KI efficiency and transgene expression in targeted integrants. This strategy can mitigate integration of fragmented transgene and unpredictable transgene silencing that were observed in recombinant cell lines generated by traditional RI method, and exploit the well‐established and simple RI‐based CLD process. Given that the recombinase‐mediated cassette exchange (RMCE)‐based CLD is increasingly being used for the development of mammalian cell lines that produce therapeutic proteins [25, 26], these KI constructs provide relatively high and stable transgene expression sites and streamlined CRISPR/Cas9‐mediated KI without the need for the selection and screening of sgRNA target sequences, suggesting as an alternative to RMCE. These artificial KI constructs can be improved by adjusting the cell‐type independent promoter and customizing the combination of elements. Thus, we demonstrated the potential of the artificial integration site as a landing pad for TI‐mediated CLD, ensuring high and stable transgene expression.

## AUTHOR CONTRIBUTIONS

Seul Mi Kim and Jae Seong Lee designed the experiments. Seul Mi Kim and Jaejin Lee conducted the experiments. Seul Mi Kim and Jae Seong Lee wrote the manuscript. All authors read and approved the manuscript.

## CONFLICT OF INTEREST STATEMENT

The authors declare no competing financial interest.

## Supporting information

Supporting Information
