# Supplementary material for: Implementation of ubiquitous chromatin opening elements as artificial integration sites for CRISPR/Cas9‐mediated knock‐in in mammalian cells
Source: Eng Life Sci. 2023 Mar 9;23(4):e2200047. doi: 10.1002/elsc.202200047 (PMC10071570; doi:10.1002/elsc.202200047)
Supplement: Supplementary file 1 — Supporting Information [file ELSC-23-e2200047-s001.docx]

**Supplementary Material**

# Implementation of Ubiquitous Chromatin Opening Elements as Artificial Integration Sites for CRISPR/Cas9-Mediated Knock-In in Mammalian Cells

Seul Mi Kim^1^, Jaejin Lee^1^, Jae Seong Lee^1,*^

^1^Department of Molecular Science and Technology, Ajou University, Suwon 16499, Republic of Korea

| **Section, figures and tables** | **Page number** |
| --- | --- |
| Supplementary figure S1 | 2 |
| Supplementary figure S2 | 3 |
| Supplementary figure S3 | 4 |
| Supplementary table S1 | 5 |
| Supplementary table S2 | 7 |
| Supplementary References | 8 |

**FIGURES**


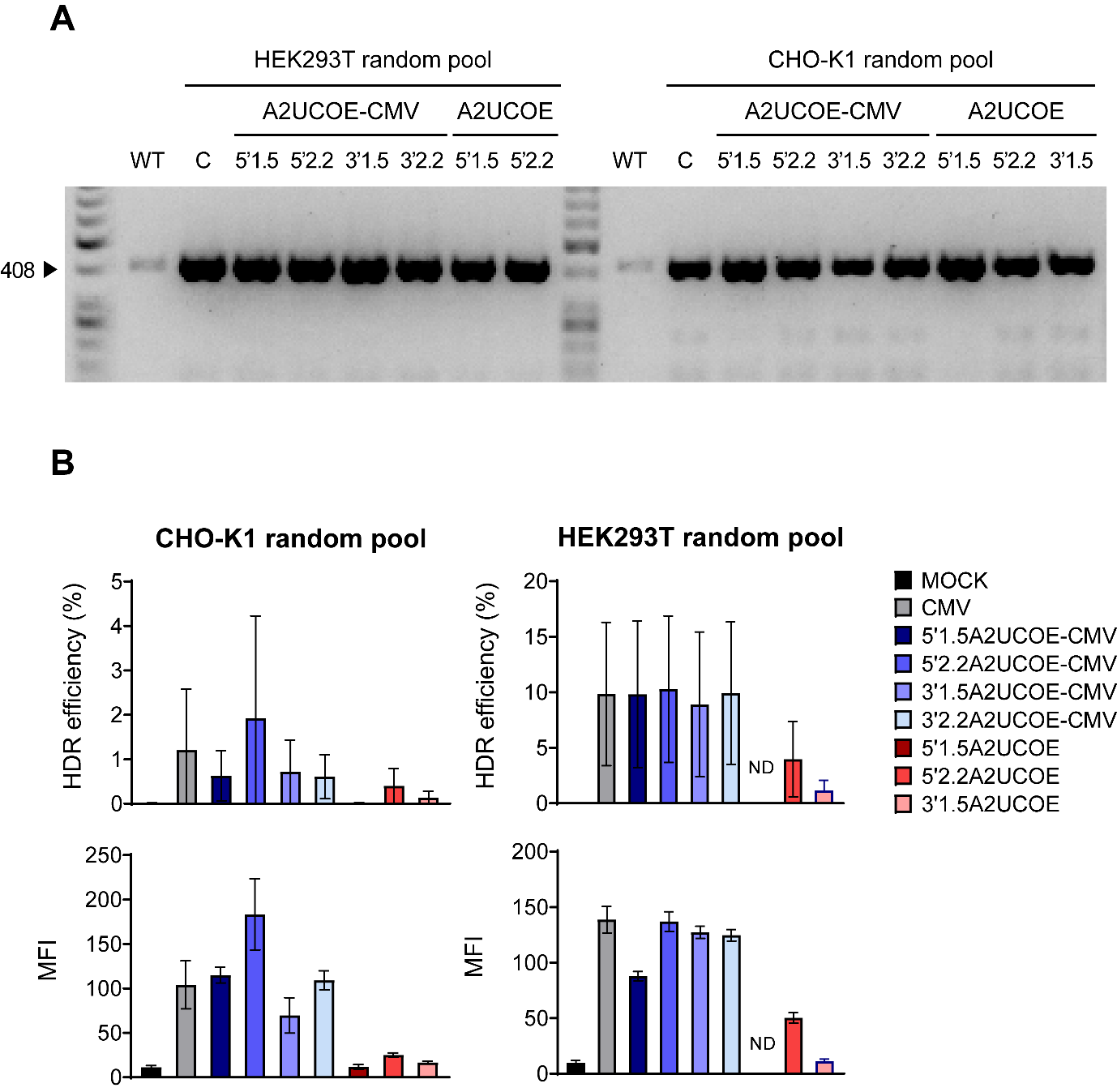


**Supplementary figure S1. Targeted knock-in in the random pools containing artificial KI constructs.** (A) PCR validation of the HDR KI of EGFP_11_ in CHO-K1 and HEK293T random pools. The primers used are listed in Supplementary table 2. (B) HDR-mediated KI efficiency and mean fluorescence intensity (MFI) of each construct in CHO-K1 and HEK293T random pools. Flow cytometry analysis was performed at 5 days after transfection. The error bars represent mean ± standard deviation of three independent experiments. ND: not detected.


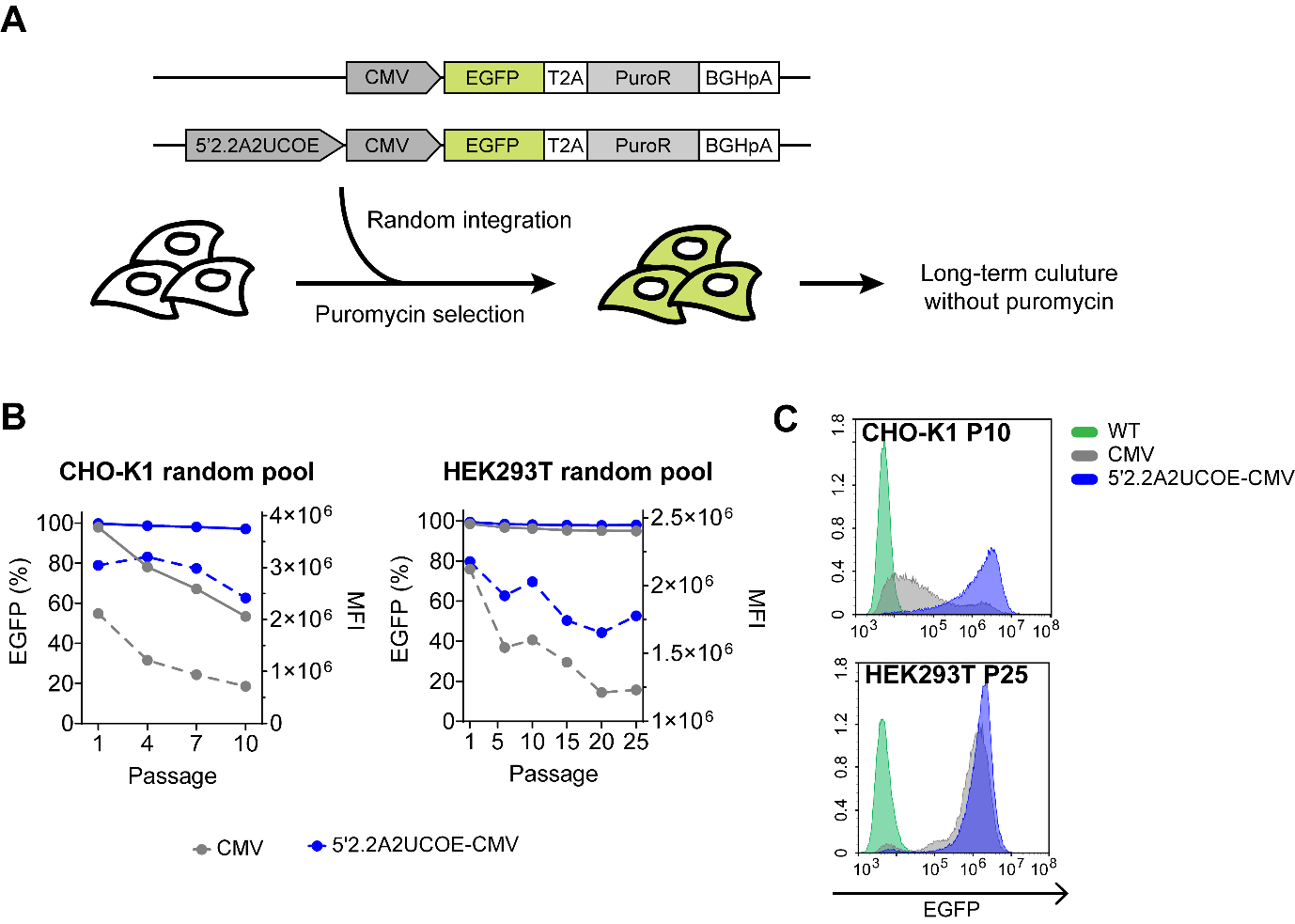


**Supplementary figure S2. 5’2.2A2UCOE-CMV-driven EGFP expression profiles.** A) Schematic illustration of the generation of CMV or 5’2.2A2COE-CMV driven full length EGFP expressing random pools and stability test of EGFP expression for long-term culture. (B) Flow cytometry analysis of EGFP expression for long-term culture. Cell pools transfected with EGFP random integration donors harboring CMV or 5’2.2A2UCOE-CMV and EGFP, were maintained in T25 flasks containing 5 mL of culture medium without puromycin. Cultured cells were passaged every 3 days at 0.2 ×10^6^ cells/mL. The EGFP-positive cell population and the expression level of EGFP protein were detected by flow cytometry. EGFP positive population (solid line) and median fluorescence intensity (MFI) (dotted line) of 5’2.2A2UCOE-CMV and CMV random pools were monitored over 10 and 25 passages, respectively. (C) Representative histogram data shown in (B) at passages 10 in CHO-K1 and passages 25 in HEK293T.

**Supplementary figure S3. Relative gene copy number of EGFP in 5’2.2A2UCOE-CMV-EGFP and CMV-EGFP random pools.** The genomic DNA from cell pellets was purified, and the relative gene copy number was analyzed using the StepOnePlus Real-Time PCR System (Applied Biosystems, Waltham, MA) and Power SYBR Green Master Mix (Applied Biosystems) as previously described.^[1]^ It was normalized to a reference cell line, which contained a single-copy EGFP in the genome.^[1]^ The error bars represent the standard deviations from technical triplicates.

**Tables**

**Supplementary Table S1.** Plasmids used in this study

| **Plasmid name** | **Description** | **Reference** |
| --- | --- | --- |
| Cas9-2A-mCherry | Cas9 2A peptide-linked mCherry without specific targeting sgRNA expression control (Addgene #64324) | [2] |
| sgEGFP_10_/Cas9-2A-mCherry | Cas9 2A peptide-linked mCherry and sgEGFP_10_ expression vector targeting EGFP_10_ and inducing DSB at after EGFP_10_ sequence | This study |
| CMV artificial KI construct random donor | Plasmid donor for random integration of artificial KI construct harboring CMV  (GOI: CMV-EGFP_10_-T2A-PuroR-BGHpA) | This study |
| 5’1.5A2UCOE artificial KI construct random donor | Plasmid donor for random integration of artificial KI construct harboring 5’1.5A2UCOE  (GOI: 5’1.5A2UCOE -EGFP_10_-T2A-PuroR-BGHpA) | This study |
| 5’2.2A2UCOE artificial KI construct random donor | Plasmid donor for random integration of artificial KI construct harboring 5’2.2A2UCOE  (GOI: 5’2.2A2UCOE -EGFP_10_-T2A-PuroR-BGHpA) | This study |
| 3’1.5A2UCOE artificial KI construct random donor | Plasmid donor for random integration of artificial KI construct harboring 3’1.5A2UCOE  (GOI: 3’1.5A2UCOE -EGFP_10_-T2A-PuroR-BGHpA) | This study |
| 3’2.2A2UCOE artificial KI construct random donor | Plasmid donor for random integration of artificial KI construct harboring 3’2.2A2UCOE  (GOI: 3’2.2A2UCOE -EGFP_10_-T2A-PuroR-BGHpA) | This study |
| 5’1.5A2UCOE-CMV artificial KI construct random donor | Plasmid donor for random integration of artificial KI construct harboring 5’1.5A2UCOE-CMV  (GOI: 5’1.5A2UCOE-CMV -EGFP_10_-T2A-PuroR-BGHpA) | This study |
| 5’2.2A2UCOE-CMV artificial KI construct random donor | Plasmid donor for random integration of artificial KI construct harboring 5’2.2A2UCOE-CMV  (GOI: 5’2.2A2UCOE-CMV -EGFP_10_-T2A-PuroR-BGHpA) | This study |
| 3’1.5A2UCOE-CMV artificial KI construct random donor | Plasmid donor for random integration of artificial KI construct harboring 3’1.5A2UCOE-CMV  (GOI: 3’1.5A2UCOE-CMV -EGFP10-T2A-PuroR-BGHpA) | This study |
| 3’2.2A2UCOE-CMV artificial KI construct random donor | Plasmid donor for random integration of artificial KI construct harboring 3’2.2A2UCOE-CMV  (GOI: CMV-EGFP_10_-T2A-PuroR-BGHpA) | This study |
| CMV-EGFP random integration donor | Plasmid donor for random integration of CMV-EGFP  (GOI: CMV-EGFP-T2A-PuroR-BGHpA) | This study |
| 5’2.2A2UCOE-CMV-EGFP random integration donor | Plasmid donor for random integration of 5’2.2A2UCOE-CMV-EGFP  (GOI: 5’2.2A2UCOE-CMV-EGFP-T2A-PuroR-BGHpA) | This study |
| EGFP_11_ HDR donor | Plasmid donor for HDR-mediated targeted knock-in of EGFP_11_ into directly after EGFP_10_ in artificial KI construct  (GOI: 5’HA(EGFP_10_ without stop codon)- EGFP_11_-3’HA(T2A-truncated PuroR)) | This study |
| T2A-TagRFP657-BGHpA NHEJ donor | HITI donor for NHEJ-mediated targeted knock-in of T2A-TagRFP657-BGHpA into artificial KI construct | This study |

**Supplementary Table S2.** Primers used in this study

| **EGFP_11_ KI validation** | | |
| --- | --- | --- |
| **Primer name** | **Component** | **Sequence (5'-3')** |
| EGFP_11__junction_fwd | Targeting to EGFP_11_ sequence | GGATCACTCTCGGCATGGAC |
| sgA2UCOE_TIDE_rev | Targeting to PuroR sequence | GGGAACCGCTCAACTCGG |
| **TIDE analysis** | | |
| **Primer name** | **Component** | **Sequence (5'-3')** |
| sgA2UCOE_TIDE_fwd | Targeting to EGFP_1-10_ sequence | TGAAGTTCGAGGGCGACAC |
| sgA2UCOE_TIDE_rev | Targeting to PuroR sequence | GGGAACCGCTCAACTCGG |

# REFERENCES

[1] Min, H., Kim, S. M., Kim, D., Lee, S. et al., Hybrid cell line development system utilizing site-specific integration and methotrexate-mediated gene amplification in Chinese hamster ovary cells. *Front. Bioeng. Biotechnol.* 2022, *10*, 977193.

[2] Chu, V. T., Weber, T., Wefers, B., Wurst, W. et al., Increasing the efficiency of homology-directed repair for CRISPR-Cas9-induced precise gene editing in mammalian cells. *Nat. Biotechnol.* 2015, *33*, 543-548.
